# Benefits of a Wearable Activity Tracker with Safety Features for Older Adults: An Intervention Study

**DOI:** 10.3390/ijerph192315723

**Published:** 2022-11-25

**Authors:** Simona Hvalič-Touzery, Mojca Šetinc, Vesna Dolničar

**Affiliations:** Centre for Social Informatics, Faculty of Social Sciences, University of Ljubljana, 1000 Ljubljana, Slovenia

**Keywords:** smart technology, telecare, physical activity, safety, older adults, mixed methods, Pillar Integration Process, GoLiveClip

## Abstract

Accidental falls and physical inactivity are important age-related issues for which smart technologies have demonstrated potential utility. This research aimed to explore the benefits of combining wearable activity monitors and telecare for older adults. A four-month interventional study was conducted between June 2021 and February 2022 in Slovenia. A purposive sample of 22 dyads of older adults aged 60 years and over and their relatives or family members used a wearable GoLiveClip device. The Pillar Integration Process was used to analyze the quantitative and qualitative data. Seven pillars emerged: (1) the use of smart technologies as a motivator for physical activity; (2) factors related to smart technology use affecting physical activity levels; (3) increased usefulness of smart technologies for users who completed the study; (4) activity monitoring as the most useful functionality of the solution; (5) the influence of technical problems on usefulness; (6) the influence of age and previous experience with smart technologies on usefulness; and (7) moderate psychological effects of smart technology use. Activity trackers were found to effectively promote physical activity in older adults, and safety features were shown to be an important part of the solution, regardless of health status or physical activity level.

## 1. Introduction

Life expectancy is increasing, and the number of people with chronic diseases is also increasing, with older people predominating [1,2]. In addition to chronic diseases, among the most common age-related issues and a major health problem in an aging society is accidental falls among older adults. Approximately one-third of people over 65 experience a fall annually [3,4,5]. Fall-related injuries, such as hip fractures, represent a significant public health problem. Older adults who fall have significantly more hospitalizations and emergency department visits than those who do not. Moreover, falls are the leading cause of injury death in older adults [3,6,7,8]. One very effective way to prevent falls is physical activity (PA) aimed at improving balance [9]. In addition to reducing falls and the risk of fall-related injuries, increased PA also contributes significantly to preventing or delaying the onset of chronic diseases and improving physical function [10,11]. By contrast, physical inactivity is among the main factors in the development of chronic diseases and is strongly associated with mortality and hospitalization [11]. A study conducted by Lee and colleagues [12] estimated that physical inactivity causes 6–10% of non-communicable diseases worldwide. According to World Health Organization (WHO) recommendations [10], at least 150 min of moderate or 75 min of vigorous PA per week, or an appropriate combination of both, is required to maintain health in older adults.

The role of smart technologies (ST) in supporting aging at home [13,14], maintaining and promoting health, and managing disease [11] is increasingly recognized in European policy. Contemporary telecare (TC) systems refer to a range of technologies, such as personal alarm systems, environmental monitors, mobility-related devices, and reminder systems [15,16,17], designed to provide greater safety and independence for the users. There is extensive evidence of the positive impact of TC use on older people (e.g., improved quality of life, increased sense of safety, greater independence, reduced social isolation and loneliness, and reduced potential consequences of falls) [18,19,20,21,22,23,24]. Wearable activity trackers (WATs) are also increasingly being used, and a number of studies have examined their impact on user behavior and well-being, particularly in the context of PA. Indeed, research has found that increasing PA has positive effects on health and psychological well-being [11,25]. WATs can be an alternative or complement to the traditional approach of increasing PA levels. WATs can increase people’s awareness of the importance of PA and engage them in monitoring their own health and habits [25]. Goal setting can also be an important motivator for increasing PA levels [26]. Several studies have found that the use of WATs can increase PA levels [11,27,28,29]. For example, a recent systematic review of systematic reviews and meta-analyses (39 studies) [30] found that WATs appeared to be effective in increasing PA in a variety of age groups. They increased PA (standardized mean difference [SMD] from 0.28 to 0.57), body composition (SMD = 0.7–2.0), and fitness (SMD = 0.3). Interventions using WATs increased users’ number of steps to approximately 1800 steps per day, walking time by approximately 40 min per day, and moderate-to-vigorous PA (MVPA) by approximately 6 min per day [30].

### 1.1. Benefits of WAT Use for Older Adults

Various studies have suggested that WATs have the potential to raise the awareness of older adults regarding their levels of PA and influence their behavior [31,32]. However, several studies that focused on older adults have found only small or moderate effects of WATs [33,34,35,36,37]. A systematic review and meta-analysis on PA levels in people aged 60 years and older [35] concluded that activity tracker interventions had a moderate effect on PA levels (23 studies; SMD = 0.55) and increased steps per day by an average of 1558, with activity tracker interventions of longer duration being more effective than interventions of shorter duration. However, no effects on quality of life were observed. By contrast, a Belgian study of older adults [37] found that, on average, participants reported that using WATs had no effect on their PA levels. The same study reported that while a significant number of participants indicated that WATs did not give them peace of mind, they also did not feel, on average, that WATs made them care about their PA and health. Half of the participants agreed that WATs could positively contribute to their overall health and have a positive impact on their level of PA, while the other half felt that WATs could not have any positive contribution to their overall health. Regarding perceived usefulness for understanding the habits and levels of PA, the study found that overall usefulness was rated as neutral, which was also reflected in the interviews, in which participants stated that listening to their own bodies was sufficient for them to become aware of the levels of PA [37].

A recent United States-based study [38] among 40 older adults who used WATs for 12 weeks found an increase in the number of steps during WAT use, as well as improvement of self-awareness and motivation for PA. They also observed that participants found WATs useful, although some concerns regarding the comfort of wearing WATs and the ease of reading visual feedback were reported. Perceived usefulness, defined as the user’s beliefs about how useful a technology is for achieving a certain goal [39], was found to be a statistically important factor in the adoption of WATs for health and fitness purposes [40]. Previous studies have identified various factors influencing the long-term use of WATs among older adults, such as initial adoption motivations, usage patterns, differences in socio-demographic factors, health status, activity levels [41], intrinsic motivation [42], age, user experience, mobile phone type, household type, perceived effect of the WAT, goal-related factors [43], perceived usefulness, and habits [31,40]. A randomized controlled trial [31] found that the device was well accepted by older long-term WAT users, who wore it on an average of 86% of possible days, and reported an overall positive experience. Furthermore, an increased sense of awareness of PA levels along with motivation was observed. The authors also found that the level of engagement with the WAT influenced the user experience, and feedback from a health professional in providing ongoing support and habits in supporting long-term behavioral change played important roles.

### 1.2. Benefits of TC Use for Older Adults

An Australian study of older people found that users of personal alarm systems (e.g., emergency calls and fall detectors) felt safer and were more active in their homes [44]. The results of a Norwegian study of 18 older adults after 5–6 months of using TC are consistent with the Australian study; older adults experienced increased safety and independence over time with the use of TC [45]. Another study from Norway, which focused on home care professionals, found that they felt that using TC protected older adults from injury and insecurity by preventing harm and providing a feeling of safety [46]. Several studies from Slovenia have confirmed these findings [47,48,49]. For example, a 12-week intervention study of 22 dyads of informal caregivers and their older relatives found that informal caregivers noted several benefits of TC for those they cared for, such as increased feelings of safety, self-efficacy, improved quality of life, peace of mind, and reassurance [47]. Another intervention study of 41 informal caregivers and 79 community-dwelling care recipients who used TC over a period of 6 to 12 months found that users reported a high level of perceived usefulness. The most commonly reported psychological outcomes of using TC among care recipients were an increased sense of control, independence, quality of life, and a sense of safety (rather than a sense of vulnerability or insecurity) [49]. Another intervention study, in which seven informal caregivers and six older care recipients tested three TC systems for eight weeks, found that confidence in timely help and safety emerged as the most important experiential outcome of using TC. This perception of safety in the eyes of users ultimately contributes to the relief of both care recipients and caregivers. In terms of timely assistance for those in need of care and their sense of safety, the key to perceived usefulness was a call center that could be alerted via a physical emergency button [48]. Similar to the findings of the Slovenian study, a US study of 21 community-dwelling older adults found that most participants perceived the usefulness of personal alarm systems in the context of falls and having someone (e.g., a call center) respond in a timely manner in an emergency [50]. In the study, the personal alarm systems led to greater independence for older adults and helped maintain their self-efficacy.

### 1.3. Aims and Research Questions

The studies described above mostly investigated the effects of WATs on PA levels as a function of the number of steps walked or the duration of a given activity [25,30], while qualitative methods were used less frequently [25], and mixed methods most rarely [37]. Furthermore, only a few studies have examined the effects of activity tracker interventions on psychosocial outcomes [30,35], and scholars have focused on the benefits of either WATs or TC for older adults, yet little is known about the benefits of ST combining both [51]. Our study therefore seeks to test ST that combines both WAT features (activity tracker and goal setting) and TC features (alarm button, fall detection, and fall risk measurement).

Within this context, the aim of our study was threefold: (1) to examine the effects of ST use on older adults’ PA levels and motivation for PA, (2) to explore the perceived usefulness of ST, and (3) to identify the psychological outcomes of using ST in older adults. By integrating the quantitative and qualitative data, we aimed to answer the following research questions:

1. What is the effect of using ST on older adults’ motivation for PA?
2. Which factors related to ST use affect PA levels among older adults?
3. Which factors related to ST use affect the perceived usefulness of ST among older adults?
4. What are the psychological outcomes of using ST among older adults?

## 2. Methods and Materials

### 2.1. Study Design and Procedure

The four-month intervention study was conducted using a quasi-experimental research design, which is often described as a non-randomized design that can be used to evaluate an intervention and focuses on causality between intervention and outcome [52]. Due to the small sample size, we used a one-group pre-test–post-test design without a control group [52]. The study used mixed methods: quantitative (pre-, during, and post-intervention questionnaire, log files—raw data) and qualitative (semi-structured post-intervention interview, open-ended questions from during and post-questionnaires). Qualitative and quantitative results were integrated and interpreted using the Pillar Integration Process [53].

A total of 22 dyads of older primary users (PU) and their relatives or family members (secondary users [SU]) were recruited in Slovenia from May to October 2021, and the intervention was conducted from June 2021 to February 2022. Both PUs and SUs completed self-report questionnaires at three time points: baseline (M0), during the intervention (M2), and after the intervention (M4). A total of 13 PU–SU dyads finished the study, and those PUs also participated in semi-structured interviews after completion. In addition, we asked, at the end of the study, whether the participants would report the daily steps they had walked during four selected weeks—Week 3 (T3), Week 6 (T6), Week 9 (T9), and Week 12 (T12)—to which 12 participants agreed. PUs who completed the study all received a gift voucher (EUR 30 in value) in appreciation of their time.

The intervention included the installation of the tested solution, GoLiveClip, on a smartphone with brief training of PUs in the use of the solution, a help desk, a “check-in” call (three times during the intervention), and four months of use of the solution by participants. The research team provided technical support at the first level (a help desk). Level 2 technical support was provided by the technical team at Gociety Solutions, the company that provides the GoLiveClip service. The project team was given access to the Crowdin translation tool to translate the text of the app into Slovenian so that the app was available to users in Slovenian.

### 2.2. Participants

Due to the restrictive eligibility criteria, purposive sampling was used to identify and select the participants. The eligibility criteria for the PUs were that they (i) had an interest in participating in the study, (ii) were aged 60 or over, (iii) owned a smartphone and had a Gmail account, (iv) were living an independent and active life, and (v) had an adult relative, family member, or friend with whom they were in regular and close contact and who was interested in participating in the study as an SU. The exclusion criteria for PUs were severe cognitive impairment (e.g., dementia) or severe physical impairment. Cognitive impairment assessment was not conducted because the recruited participants were active members of retirement associations, members of the University of the Third Age, or very active in other areas of social life and were required to use smartphones. None of them had severe dementia, because they could not be as active as they were if they did have it. Mild dementia would not pose a problem for participation in the study. Table 1 provides details of the sample for all 22 dyads that entered the study. The data presented hereafter refer only to the 13 completers. The mean age of the PUs (n = 13) was 68 years (SD = 4.5), with women being the majority (n = 9). In total, seven participants had university degrees, ten rated their health as good, and only some reported having chronic diseases, such as hypertension (n = 5) and back pain (n = 3). None of the participants suffered from diabetes or from heart or lung diseases. As required by the study, all participants owned a smartphone, but only eight rated their ability to use it as good, and the majority (n = 10) described themselves as late adopters of new technologies. They used the service being tested for an average of 126 days. The SUs (n = 13) were 42.2 years old on average (SD = 9.5), with equal proportions of men and women. All the SUs had a university degree (Table 1). 

### 2.3. Materials

The tested ST solution consisted of the GoLiveClip sensor, which communicates with the GoLivePhone app installed on the user’s smartphone [54]. ST combines features that make the user safer with WAT features (Table 2). Alerts and selected notifications were sent to the user’s emergency contact person (the SU in our study). This ST is intended to provide the user with security and a clear overview of all collected information, which can be a personal incentive for the user to become more active and achieve set personal goals more easily.

### 2.4. Measures and Data Analysis

The survey consisted of several questionnaires with standardized measurement instruments. Questions directly related to the functionalities of the tested ST and the frequency of its use were designed by the research team based on their previous research work [23,55,56]. Changes in PA were assessed using the International Physical Activity Questionnaire Short Form for Elderly (IPAQ-E) [57,58,59,60], a series of questions from the eCare Client Impact Survey (eCCIS) [61], the Log Data Inventory of Actual Steps, and interviews. The IPAQ-E questionnaire assesses PA in the past week in several domains: vigorous PA, moderate PA, walking, and sitting. For each of these physical activities, the respondent estimated the number of days of PA and the number of hours spent in that activity. Enjoyment with ST use and perceived usefulness were measured with Technology Acceptance Model (TAM) statements [39,62] on a 5-point Likert scale, where 1 meant “strongly disagree” and 5 meant “strongly agree.” The eCCIS section of the questionnaire included two statements about motivation and the ability to engage in PA. The respondent chose between answers on a 5-point Likert scale, where 1 meant “decreased a lot” and 5 meant “increased a lot.” The psychosocial impact of use was assessed using the Psychosocial Impact of Assistive Devices Scale-10 (PIADS-10) [63,64,65], which contains 10 aspects of life for which the respondents rated whether they were affected by use of the tested service on a scale from −3 (greatly decreased) to 3 (greatly increased). The PIADS-10 questionnaire was found to be reliable, with Cronbach’s alpha values greater than 0.8 at all time points (M0: 0.96, M2: 0.84, and M4: 0.94). A semi-structured interview guide for the final interview included key questions about the older users’ general experience with the tested solution, its perceived usefulness, its effect on their physical activity, and their interest in using it in the future.

Quantitative analysis. Quantitative data based on 13 PU–SU dyads were processed using IBM’s Statistical Package for the Social Sciences (version 27.0, SPSS: Armonk, NY, USA). Descriptive statistics were used. Based on the data obtained with the IPAQ-E questionnaire [58,60], we calculated the MET (metabolic equivalent), which defines the intensity of PA. One MET is defined as the resting energy expenditure that consumes 3.5 mL of oxygen per kilogram of body weight per minute (or 3.5 mL per kilogram of body weight per minute) for an average adult. Depending on intensity, PA is divided into low intensity (1–3 METs), moderate intensity (3–6 METs), and high intensity (more than 6 METs). The total value MET is the sum of METs for intense and moderate PA and walking [60]. To be classified in the “high” PA intensity group, a person must meet at least one of the following two criteria: The person has vigorous PA at least three days/week and has a total PA of at least 1500 MET-minutes/week OR the person’s total PA (a combination of walking, moderate PA, and vigorous-intensity PA) over the week is at least 3000 MET-minutes/week. Individuals in the “moderate” intensity group have moderate PA for at least 20 min on three or more days per week OR combine moderate PA and walking for at least 30 min per day on five days per week OR the sum of their total PA over the week is at least 600 MET-minutes/week. Individuals who are very low in PA and do not meet the criteria to be classified in either of the above categories are classified into the “low” PA intensity group. We calculated the average total number of steps walked weekly at the four time points for the PUs who completed the study using data stored on their smartphones on steps walked on 28 selected days.

Qualitative analysis. Semi-structured interviews were conducted using the interview guide and were recorded, transcribed verbatim, and analyzed using the ATLAS.ti 22 Windows program (ATLAS.ti Scientific Software Development GmbH, Berlin, Germany). Quality checks were performed by S.H.T. and M.S. by comparing the transcriptions with the recordings, which allowed initial familiarization with the data. We followed standard procedures for qualitative studies based on Saldaña [66] and Braun and Clarke [67]. We explored patterns of meaning across PUs in the interview data, allowing us to explore older adults’ experiences of using STs. Two authors (S.H.T. and M.S.) were involved in coding—a lead coder and a second coder who systematically cross-checked the coding. Codes were assigned to interview data by S.H.T. who ran the transcripts back and forth. Each transcript line was coded according to its meaning and content. A second coder (M.S.) reviewed the coding. Disagreements about the coding were discussed and promptly resolved. In the next step, all three authors (S.H.T., M.S., and V.D.) discussed the codes and jointly classified them into possible themes. An inductive approach was taken, which allowed the data to determine themes. Each theme was checked for internal consistency, and themes were cross-checked against each other to determine whether there were clear distinctions between themes. Themes were named based on the meaning of the theme, and described using quotations to support the analysis. Thematic analysis revealed nine themes: (1) a positive experience with the solution most pronounced among older adults who had no prior experience with activity tracking; (2) technical issues led to distrust of the solution; (3) a prevalent feeling among older users that they currently have no need for the solution; (4) safety features are important regardless of the fact that older users are still very fit and active; (5) increased self-awareness of older users as an important benefit of the solution; (6) increased motivation for PA among sexagenarians; (7) the most useful functionality is counting steps, followed by safety features; (8) the perceived usefulness of the solution is higher among sexagenarians than among septuagenarians; and (9) frequent loss of connection with the sensor as the biggest problem of the ST.

Open-ended questions from four questionnaires (M2 and M4 questionnaires from PU and SU) addressed the usefulness of ST, the effects of using ST, and the negative sides of using ST. The code scheme for the PUs’ responses to the open-ended questions was developed based on the codes developed in the semi-structured interviews. Additional codes were developed, and the initial codes were adapted to the PUs, with some existing codes deleted because no match was found. Given that the open-ended questions were narrower and less comprehensive, they only supported some results of the coding of the interviews. The themes that emerged were: (1) technical problems undermined older people’s confidence in the reliability of the solution; (2) safety features were an important part of the solution; (3) the solution increased motivation for PA in some older users; and (4) activity monitoring as the most useful feature of the solution.

**Figure 1:**
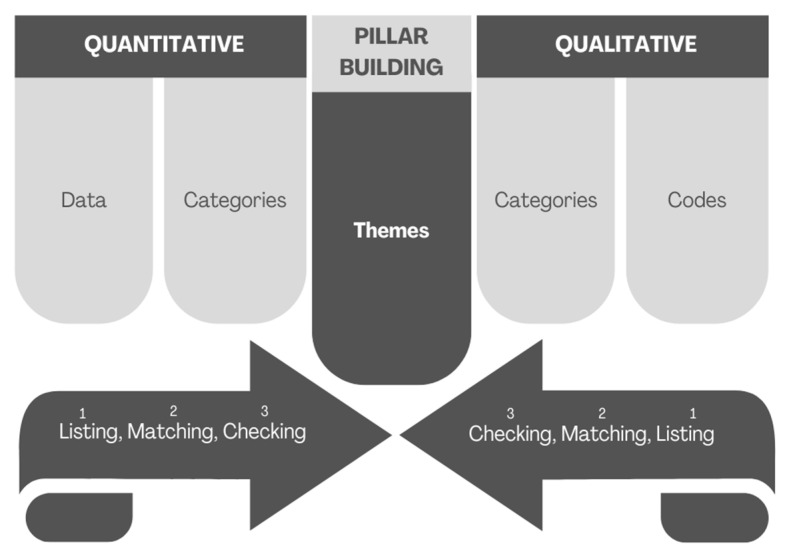
Pillar Integration Process [53].

Integrative analysis. Further analysis was based on the Pillar Integration Process (PIP), a transparent and rigorous technique for integrating and presenting qualitative and quantitative results in a joint presentation [53]. The four phases of PIP (listing, matching, checking, and pillar building) were completed sequentially after the initial quantitative and qualitative analyses were conducted separately. In Phase I (listing), two authors (S.H.T. and M.S.) used selective listing [53], referring to the studied topic. First, they listed the raw quantitative data in the quantitative data column. These data were transformed, abstracted, and listed in the quantitative categories column. In Phase 2 (matching), they matched the qualitative data to the quantitative data and the quantitative categories columns in the qualitative codes column. They identified codes and selected quotes that reflected and/or related to the quantitative data. Some qualitative data could not be matched at the end of this process. They reviewed and reorganized the data manually and matched them to the quantitative data where appropriate. In Phase 3 (checking), the data were checked for quality. The matching process was therefore reviewed independently by the third author (V.D.), who provided guidance on where changes could be made. In the final phase (pillar building), all three authors (S.H.T., M.S., and V.D.) compared and contrasted the qualitative and quantitative datasets. They aligned each row with the goals of the study. The method used is shown in Figure 1. The results of PIP are presented using tables, while the qualitative codes (participant quotes) are displayed in text form, following the example of Creaser and colleagues [68].

### 2.5. Methodological Rigur

Given the versatility of qualitative research, there is no consensus on evaluating such works. Various approaches have been proposed, with the two leading schools of thought being that of Dixon-Woods and colleagues [69], which emphasizes methodology, and that of Lincoln and Guba [70], which emphasizes rigor in interpreting results [71]. To achieve trustworthiness in the present study, the research team considered Lincoln and Guba’s [70] four criteria of credibility, confirmability, dependability, and transferability. To achieve credibility, the team used established research methods that enable the detection of the studied phenomena [71] as well as regular debriefings between the researchers during the intervention and analysis.

At all times, the participants had the option to refuse to participate in the study. They also had the option to decline to provide information on the steps recorded on their smartphones. This ensured that the participants who were included in the study were actually willing to participate and provide data [72]. They were also encouraged to be honest in their responses to the questionnaires and in the interviews. The research team also paid attention to negative case analysis [70], meaning that they refined the research questions to address all cases within the data. Finally, the credibility of the present study was enhanced by relating the results to an existing body of knowledge.

Most qualitative research studies are designed to examine a specific topic or phenomenon in a specific population and context, so generalizability of the results of qualitative research is usually not to be expected [71]. However, the application of the results of qualitative research in one situation to other similar situations (i.e., transferability) is possible. In the present study, transferability was achieved through a detailed presentation of the study context, the methods used, and the results [72].

The essence of the dependability (also called reliability) of qualitative research lies with consistency [71]. To enhance dependability, we used data triangulation—drawing research results from multiple data sources—because combining different methods balances their individual limitations and leverages their respective advantages [73]. The research design has also been described in detail so that future researchers can replicate the study [72].

Triangulation (in our case, by using the PIP method) was also important for achieving confirmability as it reduces the effect of researcher bias [72,74]. Two triangulation procedures were considered: methodological and investigator. Methodological triangulation was achieved by using a mixed methods approach, while investigator triangulation was achieved with consensus-based decision making through collaboration, discussion, and the participation of research team members with diverse perspectives [74].

The principles of validity, reliability, and generalizability were also applied in the quantitative part of the study. To ensure construct validity, the indicators and measures were carefully developed based on relevant existing knowledge, and the internal consistency reliability of the PIADS-10 was calculated using Cronbach’s alpha. Although the present study incorporated a quantitative method and thus would usually be aimed at generalizability, purposive sampling was used because it was consistent with the goals of the intervention, despite such a sample not serving the goal of generalizability [75].

### 2.6. Ethical Aspects

This study adhered to Slovenian and international codes of research ethics and the General Data Protection Regulation (GDPR) [76]. The research was approved by the Research Ethics Committee of the Faculty of Social Sciences, University of Ljubljana on 12 March 2021 (approval number: 801-2021-020/JG). All participants were informed verbally and in writing about relevant aspects of the study, such as the aims and methods, the confidentiality and anonymity of personal information, and their right to voluntarily participate in the study or withdraw at any time without consequences. Before being enrolled in the study, they gave general written informed consent to participate in the research and to have their data used in published results. In addition, the PUs who completed the study gave additional written informed consent at the end for the post-study interviews to be recorded, transcribed, and analyzed and for their data to be included in published results. Twelve of the completers explicitly agreed to provide the information stored on their smartphones about their daily steps walked during the four selected weeks.

## 3. Results

Seven pillars emerged from the integrated qualitative and quantitative results of the PIP. These were: (1) the use of ST as a motivator for physical activity; (2) factors related to ST use affecting PA levels; (3) increased usefulness of ST for users who completed the study; (4) activity monitoring as the most useful functionality of the solution; (5) the influence of technical problems on usefulness; (6) the influence of age and previous experience with ST on usefulness; and (7) moderate psychological effects of ST use. The tables in this section illustrate the integration of the findings and the resulting pillars.

### 3.1. Motivation for Physical Activity and Physical Activity Level

**Pillar 1. The use of ST as a motivator for PA**

On a scale of 1 (decreased a lot) to 5 (increased a lot), users rated their ability to engage in daily PA (3.7 in M2 and 3.6 in M4) and their motivation to do so (4.2 in M2 and 4.0 in M4) (Table 3). Both the ability and motivation to engage in daily PA increased slightly as a result of using the tested ST. This was also observed in the interviews, where we also found that increased motivation due to the tested ST was expressed more by sexagenarians (persons between 60 and 69) than by septuagenarians (persons between 70 and 79). PUs with previous experience with activity-tracking devices or apps had less positive experiences with the service or indicated that the service had no impact on them, although some in this group also spoke of increased motivation to be physically active.

*“The solution has increased my motivation to be physically active.”*

(PU03, M, 71)

*“I got insights into my activities, and the app motivates me to move more.”*

(PU15, F, 66)

*“My mother doesn’t have a problem with falls. But the app’s step-counting and other daily activity-tracking features kept her motivated.”*

(SU02, F, daughter, 39)

*“I tend to be a lazy person by nature. The sensor warns you that you haven’t walked enough and that you haven’t done enough for yourself that day. You realize that you need to take things seriously and that you need to take a little walk. From that point of view, this sensor is worth its weight in gold.”*

(PU06, F, 68)

Some PUs who had positive experiences with ST in general did not express increased motivation. Their main interest was obtaining information about their PA:

*“I did not perceive this device as a motivator for physical activity. I carried it around casually. No, I was carrying it around with me. I mean, I didn’t think it would make me achieve more, or that I would walk more. What was interesting for me was that it gave me information about my own activities.”*

(PU02, F, 73)

Enjoyment of the tested ST use was measured on a scale of 1 (strongly disagree) to 5 (strongly agree). PUs who completed the study expressed that they had fun when using the tested ST (3.8 in M2 and 3.5 in M4) and that the process of using the tested ST was pleasant (3.7 in M2 and 3.8 in M4) (Table 3). The interviews further showed that the sense of accomplishment was particularly pronounced for PUs with no prior experience with activity-tracking devices or apps, as it was the first time they could see how physically active they were. This gave them a sense of enjoyment and accomplishment, which turned out to be an important motivator for PA:

*“Based on the results that the app has shown me, my daily activity level is consistently above average, according to the WHO. This is interesting and important for my confidence and encouragement to continue to be active.”*

(PU02, F, 73)

*“I now have an overview of my activities, daily, weekly, and monthly … I am surprised that I am doing so many kilometers. I have insight into calories burned in all my activities, steps, and kilometers walked, and how many kilometers per hour I do on average.”*

(PU14, F, 62)

**Pillar 2. Factors related to ST use affecting PA levels**

**Figure 2:**
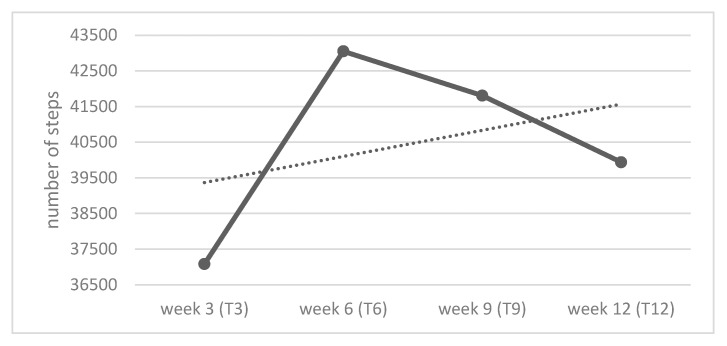
Total average number of steps per week at four time points (n = 12). Note: Out of 13 participants who completed the study, 12 provided the information on the daily steps walked during the four selected weeks.

The qualitative and quantitative data showed that several factors related to ST use affected the PA levels of PUs (e.g., novelty effect, increased self-awareness of PA, technical problems, and limitations of the tested ST). For all participants who completed the study, monitoring their own activity level was moderately to very important. The ability to ask a relative or family member for help was an important feature for 9 of 13 participants. Participants’ average number of weekly steps (Figure 2) increased rapidly at the beginning of the study, peaked in the sixth week of use, and then began to decline. Compared with the first measurement point, the average weekly total number of steps increased by 2855 steps (+7.7%) in week 12. On average, a single study participant took 24% more steps in week 12 than in week 3.

**Figure 3:**
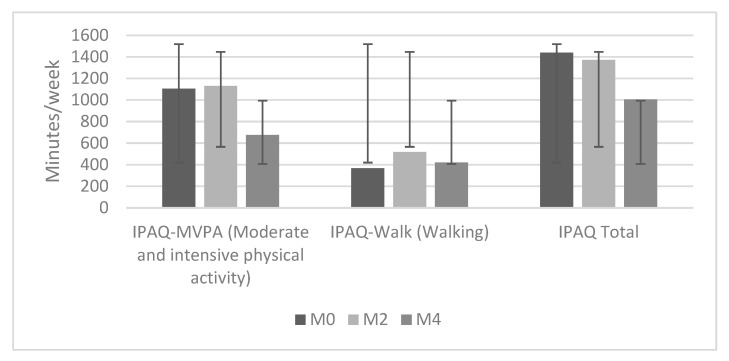
Self-assessment of weekly PA (in minutes) at the start of the intervention (M0), after two months of participation (M2), and at the end of the intervention (M4) (n = 13).

Self-assessment of PA levels showed a significant decrease in IPAQ-MVPA value (The IPAQ-MVPA value combines self-rated moderate (e.g., moderate cycling, cleaning, carrying lighter loads, raking the garden) and vigorous (e.g., carrying and lifting heavier loads, hoeing the garden, jogging, aerobics, fast cycling) physical activity in minutes per week.) during the 4-month intervention, although a small increase was observed after 2 months of participation. A self-assessment of the time spent walking for at least 10 min to and from the desired destination (IPAQ-Walk) confirmed the findings of the log data on steps walked, as we observed an increase in the number of steps during the first half of the intervention, followed by a decrease (Figure 3).

The interviews revealed that a decrease in physical activity could be due to several factors, such as forgetting to take the sensor with them when walking (once the novelty wears off), new health problems, technical problems (loss of connection between sensor and phone), temporary loss of the sensor, holiday, or vacations.

*“When you go for a walk, you leave the clip on the table at home, or you put the cell phone on the tray in the car and forget about it, and before you even realize you don’t have the cell phone with you …”*

(PU06, F, 68)

*“I have certain days when I go for a walk, and certain days when I don’t. In between, I was also sick, so I didn’t go for a walk. In the meantime, at the turn of the year, I walked quite a bit, but then I lost my sensor. So I looked for it, and it wasn’t there. But in the end, I found it. Anyway, these things happen.”*

(PU14, F, 62)

*“And then came Christmas and New Year’s vacations. So we were at home and went for walks much less.”*

(PU07, F, 77)

*“I am less active because I have health problems. That’s true. But I do exercise in the morning, every morning. And I make these 1000 movements. But the sensor does not detect these movements.”*

(PU06, F, 68)

*“In the past, there were no problems. But lately, there have been problems with the sensor. When I leave the house, I put it in the usual place in my pocket, always in the same place. Before it recorded all my steps, now hardly any steps on the whole 12-km route.”*

(PU07, F, 77)

*“I’ve been in Pokljuka for two weeks now. And I haven’t used it at all.”*

(PU13, M, 64)

Whereas in M2, 4 out of 13 SUs responded that the statement that their PUs were more physically active as a result of using the tested solution is somewhat or completely true, at the end of the intervention, 6 of the SUs reported this outcome (2.8 at M2 to 3.1 at M4) (Table 3). The interviews revealed that sexagenarians or PUs who mentioned the positive effects of use emphasized greater awareness of their PA levels.

*“I mean, it makes you think even more about the importance of being physically active. Definitely a positive influence. // I mean, it was interesting data anyway. I was measuring this activity, not that I was now increasing physical activity by I don’t know how much. I was just interested in how much I actually did.”*

(PU12, F, 70)

*“At first I thought it might be a bit distracting, that it would be a bit of a commitment, but then after a week, or I would say 14 days, I just really liked it so I was happy to track my steps, calories, and then kilometers every day.”*

(PU15, F, 66)

### 3.2. Perceived Usefulness of ST

**Pillar 3. Increased usefulness of ST for PUs who completed the study**

On a scale of 1 (not at all useful) to 5 (very useful), the perceived usefulness of the tested solution increased for PUs who participated in the intervention until the end (3.7 in M2 to 3.9 in M4), whereas SUs perceived the solution as less useful for PU at the end of the intervention (3.9 in M2 to 3.3 in M4) (Table 4). The perceived usefulness of the tested ST was also expressed during the interviews and in the open-ended questions.

*“It suits me. To me, it’s like just put it in your pocket, attach it somewhere on your clothing … I mean, this sensor is really, I have to say, really cool. It’s really very convenient”.*

(PU13, M, 64)

*“It is useful because my father knows how to use such technology”.*

(SU09, F, daughter, 43)

The perceived usefulness of ST was considered from different angles: tracking one’s PA and greater awareness of how physically active one is, encouraging a higher level of PA, and ensuring the safety of PUs.

*“It was useful because it encouraged me to move …”*

(PU03, M, 71)

*“It’s useful for checking steps, calories burned, and how many minutes you have been active.”*

(SU01, F, daughter, 48)

*“All of the activity monitoring features that were included in the solution were useful, if not otherwise, for her to monitor her physical activity a little more.”*

(SU01, F, daughter, 48)

*“I did not fall during the intervention, and I’m still in pretty good shape. So, it gives me a greater sense of safety and is a great motivator to keep moving.”*

(PU12, F, 70)

*“I would say the most important thing is that I can actually tell someone that I am in trouble in the simplest way. At the touch of a button hanging around my neck, not crawling to the phone to look for a phone that I forgot I had, that I do not know where it is.”*

(PU08, M, 69)

**Pillar 4. Activity monitoring as the most useful functionality of the solution**

Halfway through the study, PUs perceived activity monitoring (M = 4.1) and setting activity goals (M = 3.7) as the most useful functionalities of the tested solution. At the end of the study, activity monitoring remained perceived as the most useful (M = 4.0), followed by being able to send help requests to loved ones and calculating fall risk assessments (both with M = 3.2). Activity level monitoring was the most frequently used function throughout the study, as 9 of 13 PUs used this function (almost) daily at M2 and M4 (Table 4). The interview data supported these findings. Individuals who had positive experiences using the tested ST or who mentioned positive results from using the tested ST considered activity-level monitoring, especially the number of steps, and safety-related features, especially sending a request for help to a relative or family member, to be the most useful features.

*“Even when I go for a long walk alone, I feel safe because I am connected to my daughter, who would get a message if I fell. You can move more freely because you know that if you have a problem, you are connected to someone who will help you.”*

(PU16, F, 62)

*“For me, it was nice to measure how many steps I take to see how active I am.”*

(PU04, F, 62)

**Pillar 5. The influence of technical problems on usefulness**

It turned out that the tested solution did not work equally well on all smartphones, and that the frequent loss of connection between the app and the sensor in some cases undermined the confidence of PUs in the solution. These problems and limitations of the tested ST affected the perceived usefulness of the solution as well as PUs’ interest in its future use. When PUs were asked about problems with the tested ST in the last two months, only a very small number of PUs never had problems with the tested ST (M2: 1 and M4: 3). Having problems less frequently than twice a week was reported by three PUs at M2 and seven PUs at M4. At both measurement points, five PUs reported experiencing technical problems at least three times per week (Table 4). In the interviews, only half of the PUs found the tested ST suitable for monitoring activities. They placed the most value on the ability to send a request for help to a relative or family member, assuming that the problems of disconnection would be eliminated and the solution would work properly. They mentioned several problems that led to a negative experience with the tested ST and were skeptical about its usefulness in terms of inaccuracy of the measured data (steps taken were not counted continuously), the occurrence of false falls alarms due to over-sensitivity of the sensor, and loss of connection between the sensor and the app. The main drawback was the ability to record activity only when walking but not when exercising, swimming, or cycling. Lastly, PUs indicated that a sense of security was important to them, but not all of them got it with this solution.

*“You have to have your phone with you at all times, which is more difficult if you live in a house. A fall can happen if the phone is not within the reach of the device that detects the fall.”*

(SU12, F, partner, 69)

*“I am not used to having my cell phone with me all the time. That’s what bothered me the most, that I always have to have my cell phone with me.”*

(PU02, F, 73)

*“Inside the house I have to constantly check the connection, outdoors, while walking, sometimes it works, sometimes it shuts down under the same conditions. I cannot imagine what it would be like if I really needed it when I fall.”*

(PU07, F, 77)

*“The solution is not useful because there are false alarms, and it does not provide a sense of safety.”*

(PU12, F, 70)

*“False alarms that a person has fallen are too frequent, so there is a risk of not responding to the alarm because it is just another false alarm.”*

(SU12, M, partner, 69)

**Pillar 6. The influence of age and previous experience with ST on usefulness**

Data from M2 and M4 showed that the perceived usefulness of the tested ST was higher among sexagenarians (4.4 in M2 and 4.4 in M4) than among septuagenarians (2.9 in M2 and 3.5 in M4). In addition, the usefulness of the tested ST was perceived differently by PUs with (M = 3.9) and without (M = 4.2) prior experience with activity-tracking solutions (Table 4). For PUs who had no prior experience with WAT, the solution was interesting because it gave them insight into their activities and encouraged them to be active. They all cited better knowledge and awareness of their own PA levels as a benefit of ST use. The most negative experience was the frequent loss of connection between the app and the sensor. They felt that if the loss of connection problem could be fixed, the tested solution would be best for notifying relatives or family members in the event of a fall. PUs with previous experience with activity-tracking devices or apps had fewer positive experiences with the solution. Some of them felt that they did not need the tested ST because they had another app or device that better suited their needs. In addition, some users indicated that using the tested ST made them feel safer and more aware of their own activities.

*“It was useful because it encouraged me to improve. I reviewed all my physical activity results to see what I could do better.”*

(PU08, M, 69, no previous experiences with WAT)

*“Continued use would be an unnecessary expense for me for now. I have a sports tracker for activity tracking that meets my needs. The device would be useful if either of us were bedridden.”*

(PU03, M, 71, experiences with WAT)

*“You see, if the solution remains as it is, I will not buy it. I already have a smartwatch that tells me the exact steps that I walk. Maybe one day, I will buy the SOS phone.”*

(PU07, F, 77, experiences with WAT)

*“Well, I think that maybe I’m a little bit too young, too active in terms of what the device can do, for it to really help me and encourage me, or for me to feel like it would really protect me.”*

(PU01, F, 70)

*“This solution is less useful for older adults who do not own smartphones.”*

(SU09, F, daughter, 43)

### 3.3. Psychological Outcomes of ST Use

**Pillar 7. Moderate psychological effects of ST use**

At the beginning of the intervention, users were asked about the expected impact of using the service on their lives using the PIADS measurement instrument with a scale from −3 (greatly decreased) to 3 (greatly increased). Expectations regarding the effect of ST on participants’ lives at baseline were slightly higher than the actual effect during testing. The overall effect rating of ST use did not change during use (0.6 in M2 and M4). A moderately positive effect of ST use on various aspects of the PU’s life was observed. In PUs’ opinion, the use of ST had the greatest impact on eagerness to try new things. Quality of life and independence were slightly increased as a result of using TS. In M4, users were additionally asked about their sense of safety, which they perceived to have increased as a result of using the ST (Table 5 and Table 6).

In the qualitative part of the study, we found that only those who had positive experiences with the service reported an increased feeling of safety when moving around, especially outside the home, as the most important psychological effect of using the tested solution. They perceived this feature as important, even though they had no history of falls. However, they were aware of their vulnerability, especially when walking alone, and wanted to feel safe.

*“When you walk alone, you have a phone with you, but you never know if you would be able to make a call, do you? With this solution, my safety has definitely increased.”*

(PU 01, F, 70)

*“Even when I go for a long walk by myself, I feel safe because I am connected to my daughter, who gets a message if I fall. You can move more freely because you know that if you have a problem, you are connected to someone who will help you.”*

(PU14, F, 62)

*“For a person walking alone on forest trails, it’s worth a lot to know where they are if they need help.”*

(PU13, M, 64)

*“We both have a better feeling; especially if she might actually need help, she knows that someone will be informed immediately.”*

(SU15, F, daughter, 43)

SUs also pointed out the importance of the service’s safety features. For them, the most important thing was to know that if a potential adverse event occurred involving the older relative or family member, they would be notified immediately.

*“We both have a better sense of safety, especially when she really needs help, and she has a better sense that someone will be notified right away.”*

(SU13, M, son, 29)

*“Even if my mom pressed the button “by mistake/for fun”, I always reacted with great interest, as if it was a serious case. Great, because I get a text message with the location.”*

(SU08, M, son, 43)

Although the PIADS scale did not identify any negative psychological effects of using the tested ST, the negative effects of using the tested ST were mentioned in the interviews and open-ended questions. Some PUs also mentioned some negative psychological effects of using the tested ST, such as increased anxiety, worry, stress, and feeling bothered by the solution.

*“For me, the clip is a device that makes me constantly afraid of losing the connection.”*

(PU06, F, 68)

*“I am not used to having my phone with me all the time. That’s what bothered me the most that I always have to have my phone with me.”*

(PU02, F, 73)

*“It brings me stress—I have to take care of both devices to keep them charged. It’s an extra worry for me. Also, it’s another stressor to have the phone with you or make sure it’s nearby.”*

(PU06, F, 68)

*“I was walking around, and I was a little afraid of losing the sensor by accident … That was what I was most afraid of.”*

(PU13, M, 64)

*“I go a few steps from one room to another, like the kitchen, and it gets disconnected. In the beginning, I had to keep turning it on, and that was nerve-wracking, I have to admit.”*

(PU13, F, 77)

## 4. Discussion

The PUs in our study were independent and physically active in their daily lives. Some of them had previous experience with activity-tracking devices, whereas, for others, this intervention was their first contact with STs of this kind. The participants also differed in their levels of PA. Our study found some benefits of using ST for older users, such as increasing PA levels and motivation, and positive psychological outcomes. However, these benefits were moderate and not present for all users. This could also be due to the particular nature of the technology and some reported technical problems. Nevertheless, some PUs found the tested ST useful.

First, the study provided evidence of moderate effects of ST use on older adult’s motivation for PA. These positive usage effects were particularly pronounced among PUs who had no prior experience with activity monitoring devices or apps. The PUs in our study predominantly reported their intrinsic motivation to be physically active. Intrinsic motivation refers to engaging in an activity out of authentic interest and enjoyment of the activity itself, whereas extrinsic motivation refers to engaging in activities that are externally rewarded [77]. Given the nature of our study, in which no external motivator (e.g., some authority has ordered PU to be physically active) was behind the tested ST, the primary role of intrinsic motivation is not surprising. A US study also suggests that the long-term use of activity trackers depends primarily on intrinsic motivation and the positive emotional rewards users receive after achieving their goals [42]. Intrinsic motivation can also “bring inherent satisfaction and feelings of enjoyment, accomplishment, and excitement” [42] (p. 10), which was also observed in our study, based on the comments from some PUs that the information on their PA gave them feelings of enjoyment and accomplishment. The tested ST provided them with information about their PA, but did not motivate each of them to be more active. In addition, our study showed that PUs who had no prior experience with activity-tracking devices had more positive experiences with the solution and were more aware of and engaged in their PA. These findings are consistent with a US study [42] that found that activity tracker non-users who were offered an activity tracker to use for several weeks reported that using the device helped them increase their PA levels.

Second, self-assessment of PA and log data on PA levels showed little increase in the number of steps taken. These data could also be due to the occasional loss of connection between the sensor and the app and the resulting small number of steps or the season (winter) in which the intervention study predominantly took place (lower PA than in other seasons). A number of studies have found that the use of WATs stimulates PA levels [11,27,28,29] in participants, including patients with a variety of chronic health conditions (e.g., diabetes, elevated blood pressure, and cardiovascular disease) who were managed by health care professionals, resulting in more disciplined and informed users. A German study among 80 older adults found a small effect on moderate to vigorous physical activity (MVPA) after the intervention (+19 min per week), and a medium-sized effect on the number of steps (+1317 per day) compared to the baseline values. In the follow-up, the intervention effect was still significant for the number of steps (+844 per day), whereas MVPA dropped back to the baseline values [78]. Furthermore, another US study [38] reported an average increase of 900 steps per day among 40 older adults who used WATs for 3 months. Our study results are more in line with the results of other previous studies that focused on older adults [33,34,35], which found low or moderate effects of WATs on older adults’ PA levels. Studies [37,43,79] have also found that users of WATs are often highly motivated at baseline, but this motivation declines as use progresses, which was also found in our study. Indeed, the “novelty effect” of using the service began to wane after six weeks. According to Ridgers and colleagues [79], WATs can be useful in raising an individual’s self-awareness of their PA, but additional approaches are likely needed for long-term change.

In addition to the novelty effect, our study found that several factors related to ST use affected the PA level among PUs (e.g., increased self-awareness of PA, technical problems and limitations of ST, holidays, or vacations). PUs who participated in the study had a greater sense of self-awareness due to their use of ST, which affected their PA levels. This finding is consistent with a 12-month randomized controlled trial [31] that reported a link between the sense of awareness of PA levels and PA levels of 20 older adults with chronic conditions who used WATs in combination with feedback from health care professionals. This relationship between self-awareness and improved or maintained PA behaviors has also been reported in other studies [38,80,81,82,83], although increased awareness does not necessarily lead to increased motivation, as observed in our study and in previous research [31]. A Canadian qualitative study of 32 participants who tested five WATs [80] found that the greatest benefit of the WATs was that they helped participants become more aware of their PA levels, which is consistent with the findings of our study. In addition, they acknowledged the importance of the perceived usefulness of the devices in lifestyle choices, leading to increased intention to use and ultimately adoption of the technology.

The chronic conditions of WAT users were not a focus of the present study, despite such conditions being shown to affect WAT use in other studies [11,41,84,85,86], because most of the participants did not have chronic conditions that might limit their use of WAT, such as arthritis or chronic obstructive pulmonary disease, which have been associated with a lower likelihood of long-term use [41,84,85,86]. Similarly, a review of randomized controlled trials evaluating WAT interventions [11] showed that participants with chronic respiratory or cardiovascular diseases had significantly smaller increases in PA than did populations with other chronic conditions. Third, perceived usefulness was rated high by our study participants who completed the study, particularly in terms of tracking PA levels, improving self-awareness of one’s PA, encouraging more PA, and ensuring safety. According to a US study, older adults aged 65–74 were 1.4-fold more likely to use a WAT than those 85 and older [87]. In our study, sexagenarians reported more positive experiences and increased motivation for PA than the other participants. Since the level of engagement with the activity tracker influences the user experience [31], it is important to note that our study found that most participants who used the solution until the end of the study used the feature daily or almost daily. This indicates their interest in a technical solution and the potential for future solutions that offer activity monitoring with included safety features. Indeed, as Liu and colleagues [28] found, wearing WAT daily increases the likelihood of its long-term use.

In our study, the tested WAT was shown to be not always reliable and to have technological drawbacks that affect the perceived usefulness of the ST. Further, in a Swedish study of community-dwelling older adults, some experienced technical problems with the WAT that prevented them from collecting PA feedback [88]. Another study based on a general population sample found that nearly three-quarters of participants stopped using activity trackers 100 days after the start date, with most dropouts due to technical errors and loss of activity trackers [43]. A Canadian study of 20 community-dwelling older adults [82] also found that WAT acceptance may be affected by WAT’s heavy reliance on smartphones for visualizing data, which was also the case in our study. Participants expressed concerns about perceived ease of use through fear of forgetting, losing, or breaking the smartphone, as well as inconvenience of carrying them, which influenced their future intention to use it. The inconvenience of carrying smartphones was also expressed by the PUs in our study.

Finally, our study found a moderate positive effect of ST use on quality of life, independence, and other aspects of life. In both the qualitative and quantitative studies, the feeling of safety was mentioned by some PUs as a positive result of using ST. As in other studies [19,47,48,89,90,91], we confirmed that features that ensure user safety are an important functionality of ST, regardless of the health status of the older user. The importance of safety features and the benefits they bring to older adults (the feeling of being able to easily notify someone when needed) were also expressed by secondary users in our study. Similar results were observed in several other studies [15,47,48,92,93]. Our study also observed that the increased feeling of safety was pronounced only in those PUs who had a positive experience with the tested ST. PUs with negative experiences, who mentioned various technical problems with the solution and consequently its unreliability, indicated that the problems with the solution made them feel more stressed or that the service was of no value to them because they could not trust it to be of real help in a potential crisis situation. This was highlighted not only by older PUs who had negative experiences but also by SUs who said they stopped responding to notifications (because of their frequency). The negative psychological impact of incompletely functioning STs has also been noted in other studies [24,47].

### Strengths and Limitations

The strengths of this study lie in the use of mixed methods and the integration of the quantitative and qualitative findings using the PIP analytical technique. This allowed for a more comprehensive view of the topic studied and strengthened the validity and reliability of the results. Another strength is the involvement of PUs and SUs in capturing perceptions of the benefits of STs. Important limitations of this study are the small sample size and the large dropout rate among the participants. The latter is probably and partially due to technical problems with the tested solution. Although we selected an award-winning Dutch service for the test, it did not prove most reliable in the Slovenian environment, mainly due to its incompatibility with the smartphones owned by some of the PUs. The biggest problem was the frequent disconnection between the sensor and the app, which could not be resolved. Although data saturation was achieved in the qualitative analysis, the small sample size was only sufficient for an initial assessment of the effects of using the service. The smart solution also required the use of a smartphone and a Gmail account—inclusion criteria that influenced the sample of participants.

Furthermore, responses could have been influenced by the Hawthorne effect [94], which states that participants may change their behavior when participating in a study and may respond with answers they believe are consistent with what the researchers want to hear. The study participants were informed prior to the intervention that the researchers would not have access to the information collected on their smartphones and that it was up to the participants to report the results as they observed them. The decision to ask participants if they would be willing to share information about their daily steps was made only at the end of the study because the dropout rate was high and additional data were needed to add weight to the study. Since the Hawthorne effect refers to behavioral changes resulting from prior awareness of being the subject of an experiment, we believe that the effect was small because the original awareness was that the data would not be shared. The results of the interviews and the questionnaires at the three time points were consistent with the actual log data on the smartphones that the participants willingly provided—but were explicitly not required to do so—further suggesting little or no impact of the Hawthorne effect. Given the limitations of the study, it would be useful in future to test more smart solutions in different national contexts, recruit a larger number of users, and conduct randomized controlled trials of possible smart solutions. 

## 5. Conclusions

This study provides insights into the benefits of WATs with safety features for older adults by outlining PUs’ and SUs’ perceptions of an ST, its perceived usefulness, and the effects of its use. The results of this study, similar to those of other studies [42], suggest that activity trackers may be an effective technology for promoting physical activity in older adults, especially in those who have never tried them. Furthermore, our study demonstrated the importance of safety features for older adults, regardless of their health status or PA level. Although the older participants were still physically active, they were aware of the potential dangers of falls and therefore desired an added sense of security that fully functional technological solutions could provide. Thus, WATs with safety features may be a plus for many potential older adults. In addition, it is important to note that such a smart technological solution should be unobtrusive, easy to use, and preferably not directly connected to a smartphone, as older adults are not accustomed to carrying their phones with them at all times. It is also important to consider the needs of the users and to pay attention to the usefulness of such a solution, especially knowing that perceived usefulness influences the acceptance [95] of the technology.

## Figures and Tables

**Table 1:** Baseline characteristics of all participants, participants who completed the study, and participants who dropped out.

	**Total (n = 22)**	**Completers (n = 13)**	**Drop-Outs (n = 9)**
**Primary users**			
Age (years), M ^1^ (SD) ^2^ (min–max)	68.95 (4.0) (62–77)	68.08 (4.5) (62–77)	70.22 (2.9) (67–75)
*Gender*, *n* (%)			
Male	5 (22.7%)	4 (30.8%)	1 (11.1%)
Female	17 (77.3%)	9 (69.2%)	8 (88.9%)
*Education level*, *n* (%)			
Two- or three-year vocational school	2 (9.1%)	0	2 (22.2%)
Four-year secondary school, high school	7 (31.8%)	2 (15.4%)	5 (55.6%)
Higher education	4 (18.2%)	4 (30.8%)	0
College, university or academy or more	9 (40.9%)	7 (53.8%)	2 (22.2%)
*Health*, *n* (%)			
Very good	1 (4.5%)	0	1 (11.1%)
Good	14 (63.6%)	10 (76.9%)	4 (44.4%)
Fair	6 (27.3%)	2 (15.4%)	4 (44.4%)
Poor	1 (4.5%)	1 (7.7%)	0
*Intensity of PA—*(*MET*^3^—*minutes*/*week*) (*n* = 20) ^4^			
High, n (%), M (SD)	12 (54.5%), 10,019.3 (5443.6)	8 (61.5%), 10,503.7 (6348.8)	4 (57.1%), 7597.7 (3157.0)
Moderate, n (%), M (SD)	7 (31.8%), 1592.3 (727.0)	4 (30.8%), 1081 (297.3)	3 (42.9%), 2274 (354.9)
Low, n (%), M (SD)	1 (4.5%), 558.0 (0)	1 (7.7%), 558.0 (0)	0
*The ability to use a smart phone*, *n* (%)			
Very good	4 (18.2%)	2 (15.4%)	2 (22.2%)
Pretty good	9 (40.9%)	6 (46.2%)	3 (33.3%)
Neither good nor bad	9 (40.9%)	5 (38.5%)	4 (44.4%)
*Type of technology adopter*			
Early adopters ^5^	4 (18.2%)	3 (23.1%)	1 (11.1%)
Late adopters ^6^	18 (81.8%)	10 (76.9%)	8 (88.9%)
*Family relation to the secondary user*, *n* (%)			
Parent	14 (63.6%)	10 (76.9%)	4 (44.4%)
Partner	4 (18.2%)	2 (15.4%)	2 (22.2%)
Other	4 (18.2%)	1 (7.7%)	3 (33.3%)
The average days in the study, M ^1^ (SD) ^2^	98.5 ^7^ (41.4)	125.9 (3.7)	62.9 ^8^ (6.1)
	**Total (n = 22)**	**Completers (n = 13)**	**Drop-outs (n = 9)**
**Secondary users**			
Age (years), M ^1^ (SD) ^2^	46.83 (14.7)	42.2 (9.5)	58.8 (17.5)
*Gender*, *n* (%)			
Male	12 (54.5%)	7 (53.8%)	5 (55.6%)
Female	10 (45.5%)	6 (46.2%)	4 (44.4%)
*Education level*, *n* (%)			
Finished primary school	1 (5.6%)	0	1 (20%)
Upper secondary vocational or general education	1 (5.6%)	0	1 (20%)
Higher education	1 (5.6%)	0	1 (20%)
College, university, academy or more	15 (83.3%)	13 (100%)	2 (40%)

Note. ^1^ M = mean; ^2^ SD = standard deviation; ^3^ MET (metabolic equivalent); ^4^ Two were excluded (unrealistic numbers, one person had a medical procedure). ^5^ The “early adopters” were respondents who responded that they tried technologies as soon as they appeared on the market before others tried them. ^6^ The “late adopters” were respondents who responded, “I wait until technologies are in general use before I start using them” and “I am one of the last to adopt an innovation”. ^7^ The average number is the average of participants who successfully completed the study and those who had a successful installation (n = 20). ^8^ Only those who had successfully installed or started using the tested solution before dropping out of the study were included in the calculation (n = 5).

**Table 2:** Summary of services supported by the tested solution.

	Input	Output	Purpose
**TC Features**			
Self-triggering alarm button	Triggering alarm by pressing the button	Sending an alert to the enlisted emergency contacts	Real-time notification in case of emergency
Automated fall detection	Detected falling incident	Sending an alert to the enlisted emergency contacts	Fall detection; real-time notification in case of potential falling incident
Fall risk measurement	Personal data (age, weight, height) and recorded data (walking asymmetry, walking speed, number of steps, distance walked)	Notification on user’s smartphone and alert sent to the enlisted emergency contacts	Assessment of fall risk on a scale from 1 to 10; historical trending analysis and informing when the risk of a falling incident goes up
**WAT Features**			
Goal setting	Entering goals regarding the activity (WHO recommendation—at least 150 min of moderate physical activity per week OR daily steps goal OR daily calories burned goal)	Goals attainment (virtual medals)	Motivation to become more active or to reach certain specific goals
Activity tracker	Personal data (age, weight, height), integrated sensors	Number of steps, number of calories burned, total time of activity, average speed of movement, distance walked, MET (metabolic equivalent)—value of the activity	Clear overview of all collected information on user’s physical activity

**Table 3:** A joint display of the connections between the quantitative and qualitative data arising from the study—Pillars 1 and 2.

Quantitative Data	Quantitative Categories	Pillar	Qualitative Categories (Themes Derived from the Thematic Analysis)
**M2 and M4 PU questionnaire** Motivation to engage in PA due to the use of the solution M2: M = 4.2 (SD = 0.7); M4: M = 4.0 (SD = 0.7) *Scale: 1 = decreased a lot … 5 = increased a lot*	PUs’ motivation for PA	**Pillar 1. The use of ST as a motivator for PA**	**Interview** STs as a PA motivator but not for all PU
			**Open-ended questions (M2 and M4 SU questionnaire)** According to some SUs, the solution increased the motivation of their older relatives or family members for PA
			**Open-ended questions (M2 and M4 PU questionnaire)** The solution increased the motivation for PA among some PUs.
No corresponding quantitative data	n/a		**Interview** Increased motivation for PA due to ST use expressed more by sexagenarians
**M2 and M4 PU questionnaire** Enjoyment with the ST M2: M = 3.8 (SD = 0.9); M4: M = 3.5 (1.3) ST usage being pleasant M2: M = 3.7 (SD = 0.9); M4: M = 3.8 (SD = 0.9) *Scale: 1 = decreased a lot … 5 = increased a lot*	Intrinsic motivation brought feelings of enjoyment		**Interview** Prevalence of intrinsic motivation to use ST among PUs (feelings of enjoyment and accomplishment)
**M2 and M4 PU questionnaire** IPAQ Walk: Increase in the number of steps during the first half of the intervention, followed by a decrease	PA levels among PU	**Pillar 2. Factors related to ST use affecting PA levels**	No corresponding qualitative data
**Log data** PUs’ average number of weekly steps increased rapidly at the beginning of the study, peaked in the sixth week of use, and then began to decline.			**Interview** Various factors affected temporal changes in PA levels
**Log data** The average weekly total number of steps increased by 2855 steps (+7.7%) in week 12.			
**M2 and M4 SU questionnaire** PU was more physically active due to use of ST M2: M = 2.8 (SD = 1.3); M4: M = 3.1 (SD = 1.3) *Scale: 1 = does not apply at all… 5 = applies completely*			No corresponding qualitative data
**M2 and M4 PU questionnaire** Importance of monitoring own PA M2: M = 4.1 (SD = 1.0); M4: M = 4.0 (SD = 0.6) *Scale: 1 = not important at all … 5 = very important*	Self-awareness of PA levels		**Interview** Relationship between an increase in PA levels and self-awareness of PA
No corresponding quantitative data	n/a		**Interview** Sexagenarians more than septuagenarians mentioned increased self-awareness of PA

**Table 4:** A joint display of the connections between the quantitative and qualitative data arising from the study—Pillars 3, 4, 5 and 6.

Quantitative Data	Quantitative Categories	Pillar	Qualitative Categories (Themes Derived from the Thematic Analysis)
**M2 and M4 PU and SU questionnaire** The perceived usefulness of ST—PUs M2: M = 3.7 (SD = 0.6); M4: M = 3.9 (SD = 0.8) The perceived usefulness of ST—SUs M2: M = 3.9 (SD = 0.7); M4: M = 3.3 (SD = 1.2) *Scale: 1 = not at all useful … 5 = very useful*	Usefulness of ST increased over time for PUs who completed the study	**Pillar 3. Increased usefulness of ST for PUs who completed the study**	**Interview** Half of PUs who completed the intervention perceived ST as useful
			**Open-ended questions (M2 and M4 PU and SU questionnaire)** Perceived usefulness expressed by some PUs and SUs during the study
No corresponding quantitative data	n/a		**Interview** ST was most useful for tracking PA, improving self-awareness of one’s PA, encouraging more PA, and ensuring safety
No corresponding quantitative data	n/a		**Interview** ST of no value to PUs experiencing various technical problems that make ST unreliable
**M2 and M4 PU questionnaire** Following on activity on (almost) daily basis: M2 and M4: 69.2% Activity monitoring was the most frequently used functionality.	Activity monitoring as most useful functionality	**Pillar 4. Activity monitoring as the most useful functionality of the solution**	No corresponding qualitative categories
**M2 and M4 PU questionnaire** Functionalities rated as the most useful—M2 Activity monitoring: M = 4.1 (SD = 1.0) Setting activity goals: M = 3.7 (SD = 1.3) Functionalities rated as the most useful—M4 Activity monitoring: M = 4.0 (SD = 0.6) Sending help to relatives or family members while being outdoors: M = 3.2 (SD = 1.7) Fall risk assessment: M = 3.2 (SD = 1.4) *Scale: 1 = not at all useful … 5 = very useful*			**Interview** The most useful functionality was counting steps, followed by safety features
**M2 and M4 PU questionnaire** Frequency of problems when using ST in the last two months a. Never: M2: 1 PU; M4: 3 PU b. Less frequently than twice a week: M2: 3 PU; M4: 7 PU c. At least three times per week: M2: 5 PU; M4:5 PU	Technical problems with ST	**Pillar 5. The influence of technical problems on usefulness**	**Interview** ST of no value to PUs experiencing various technical problems that make ST unreliable
**M2 and M4 PU questionnaire** Perceived usefulness of ST sexagenarians M2: M = 4.4 (SD = 0.7) M4: M = 4.4 (SD = 0.8) Perceived usefulness of ST—septuagenarians M2: M = 2.9 (SD = 1.2); M4: M = 3.5 (SD = 1.1) *Scale: 1 = not at all useful … 5 = very useful*	Perceived usefulness influenced by age and prior experiences with WAT	**Pillar 6. The influence of age and previous experience with ST on usefulness**	**Interview** Perceived usefulness of the solution higher among sexagenarians than septuagenarians
**M4 PU questionnaire** Perceived usefulness of ST based on previous experience with activity tracking No prior experience: 4.2 (SD = 1.0) Prior experience: 3.9 (SD = 1.1) *Scale: 1 = not at all useful … 5 = very useful*			**Interview** Differences in perceived usefulness evident between those with and without prior experience with activity-tracking solutions

**Table 5:** Aspects of life for which PUs rated whether they were affected by the use of the ST at three time points (n = 13).

PIADS	M0 ^2^	M2	M4
	M (SD)	M (SD)	M (SD)
Happiness	0.6 (0.8)	0.4 (0.7)	0.5 (0.7)
Independence	0.6 (1.0)	0.5 (0.7)	0.6 (0.8)
Self-esteem	0.6 (1.0)	0.6 (0.8)	0.5 (0.5)
Productivity	0.7 (0.9)	0.3 (0.6)	0.3 (0.6)
Quality of life	0.9 (0.9)	0.5 (0.5)	0.7 (0.9)
Sense of control	0.9 (0.9)	0.6 (0.9)	0.4 (0.6)
Ability to participate	0.8 (0.7)	0.5 (0.8)	0.5 (0.8)
Eagerness to try new things	1.2 (0.4)	1.3 (1.0)	1.3 (1.1)
Ability to adapt to the activities of daily living	1.1 (0.8)	0.5 (0.8)	0.5 (0.7)
Ability to take advantage of opportunities	1.1 (0.8)	0.9 (0.8)	0.8 (0.9)
Feeling safe ^1^	n.d. ^3^	n.d. ^3^	1.00 (0.9)
Total M (SD) Q1 Q3	0.84 (0.7) 0.40 1.0	0.60 (0.48) 0.25 0.85	0.59 (0.63) 0.05 0.85

Note. A scale of −3 (greatly decreased) to 3 (greatly increased); 0 signifies that it did not increase or decrease. Values Q1 in Q3 represent the PIADS-10 values at the lower (Q1) and upper (Q3) quartiles. ^1^ The feeling of safety is included in PIADS-26 and not in PIADS-10. It was added to the questionnaire after we noticed during a follow-up call to participants that they very often mentioned a feeling of safety in relation to the use of the ST. ^2^ At M0, the questionnaire captured expectations, while questionnaires M2 and M4 captured the effects of using ST. ^3^ No data.

**Table 6:** A joint display of the connections between the quantitative and qualitative data arising from the study—Pillar 7.

Quantitative Data	Quantitative Categories	Pillar	Qualitative Categories (Themes Derived from the Thematic Analysis)
**M2 and M4 PU questionnaire** No negative effects of using ST were found. Overall ratings of the effects of use ranged from 0.3 to 1.3 at M2 and M4. *Scale: −3 = greatly decreased … 3 = greatly increased; 0 = no decrease or increase*	Moderate psychological effects of ST use	**Pillar 7. Moderate psychological effect of ST use**	**Interview and Open-ended questions (M2 and M4 PU questionnaire** Technical issues and the demands of ST brought to some PUs negative psychological outcomes
**M4 PU questionnaire** At M4, the use of ST affected feelings of safety: M = 1.0 (SD = 0.9). The eagerness to try new things remained relatively high from the beginning to the end of the study (M4: 1.3 (SD = 1.1)). *Scale: −3 = greatly decreased … 3 = greatly increased*	Increased feeling of safety as a result of ST use		**Interview** PUs with a positive experience with ST reported an increased feeling of safety when walking around
No corresponding quantitative data	n/a		**Open-ended questions (M2 and M4 SU questionnaire)** Safety features of the ST solution important to SUs
No corresponding quantitative data	n/a		**Open-ended questions (M2 and M4 PU questionnaire)** Safety features important regardless of older users still being very fit and active

## Data Availability

The data presented in this study are not publicly available due to ethical restrictions.
